# Effects of Hygrothermal Condition on Water Diffusion and Flexural Properties of Carbon–Glass Hybrid Fiber-Reinforced Epoxy Polymer Winding Pipes

**DOI:** 10.3390/polym16233433

**Published:** 2024-12-06

**Authors:** Ying Zhao, Qiang Li, Guoqiang Zhou, Kehai Zhu, Bo Jing, Kangnan Zhu, Jiajun Shi, Chenggao Li

**Affiliations:** 1 Sinopec Offshore Petroleum Engineering Inspection Co., Ltd., Dongying 257000, China; zhaoying185.slyt@sinopec.com (Y.Z.); liqiang228.slyt@sinopec.com (Q.L.); zhougq139.slyt@sinopec.com (G.Z.); 2 Shengli Oilfield Branch Company, China Petroleum & Chemical Corporation, Dongying 257000, China; zhukehai.slyt@sinopec.com (K.Z.); jingbo907.slyt@sinopec.com (B.J.); 3 School of Civil Engineering, Harbin Institute of Technology (HIT), 73 Huanghe Road, Nangang District, Harbin 150090, China; jjshi98@foxmail.com (J.S.); lichenggao@hit.edu.cn (C.L.)

**Keywords:** carbon–glass hybrid fiber, winding pipe, hygrothermal condition, flexural properties, durability

## Abstract

Carbon–glass hybrid fiber-reinforced epoxy polymer (C-GFRP) winding pipes integrated with the advantages of light weight, high strength, corrosion resistance, and cost-effectiveness offer immense potential to mitigate corrosion issues in oil, gas, and water transportation pipelines. In this study, C-GFRP winding pipes underwent accelerated aging tests through immersion in distilled water at temperatures of 25 °C, 40 °C, and 60 °C for 146 days. Water absorption tests were conducted to investigate the water absorption behavior of only CFRP- or GFRP-side absorbed water. Bending tests were performed to assess the evolution of the pipes’ flexural properties in two directions (GFRP or CFRP in tension). The results showed that the single-sided water absorption behavior adhered to the two-stage diffusion model. The diffusion coefficient, activation energy, and 146-day water absorption were all higher for the CFRP-side absorbed water compared to the GFRP-side absorbed water. The flexural strength and modulus of C-GFRP pipes were influenced by post-curing and resin hydrolysis/debonding. Initially, the flexural strength of CFRP in tension was higher than that of CFRP in tension. After 146 days of aging, the flexural strength of CFRP in tension was lower than that of CFRP in tension. Utilizing Arrhenius theory, the long-term lives were predicted for the flexural strength at temperatures of 5.4 °C, 12.8 °C, and 17.8 °C. The predicted lives of GFRP in tension were higher than those of CFRP in tension.

## 1. Introduction

Steel pipelines serve as crucial structures for the transportation of oil, gas, and water. By 2025, the total mileage of China’s oil and gas pipeline network is anticipated to escalate to 240,000 km [1,2]. Nonetheless, these pipelines are susceptible to significant corrosion and maintenance challenges due to the combined influences of alternating humid and hot external environments, such as those encountered in oceans, lakes, and during rainy seasons, as well as prolonged exposure to hygrothermal internal conditions [3]. Fiber-reinforced polymer (FRP) winding pipes, fabricated through cross-winding and curing of resin-impregnated fibers, offer notable advantages including light weight, high strength, and corrosion resistance [4,5]. They have become potential alternatives to traditional steel pipes [6]. Commonly utilized FRP pipes include glass fiber-reinforced polymer (GFRP) winding pipes and carbon fiber-reinforced polymer (CFRP) winding pipes, etc. [7,8]. While CFRP exhibits superior corrosion resistance and strength compared to GFRP [9,10], its application is often restricted by high costs [11,12,13]. A novel carbon–glass hybrid fiber-reinforced epoxy polymer (C-GFRP) winding pipe has been developed, utilizing a CFRP winding thin pipe as the inner lining and a GFRP winding thick layer as the outer wrapping. This design aims to strike a balance between cost-effectiveness and good properties [14]. Consequently, it is imperative to investigate the property variations of C-GFRP pipes under complex hygrothermal conditions both inside and outside the pipe.

Previous research has demonstrated that the degradation of FRP materials in hygrothermal conditions is primarily attributed to water molecule diffusion [15,16]. After water molecules enter the interior of the material, the network structure of the resin polymer relaxes, leading to resin hydrolysis and fiber resin interface debonding [17,18]. It has been established that the water absorption diffusion behavior in numerous GFRP and CFRP materials adheres to Fick’s law model [19,20,21]. This model analogizes the water molecules’ diffusion to a thermal conduction process, driven by concentration gradients [22,23]. Additionally, some studies report that the water absorption diffusion behavior in some CFRP materials and C-GFRP hybrid materials follows a two-stage model [17]. According to this model, water absorption diffusion is initially governed by water molecule concentration, followed by the relaxation of the resin molecular structure in the second stage [24]. However, existing research focuses on CFRP, GFRP, or C-GFRP materials as a whole. When the fiber volume content is equivalent, the water absorption diffusion coefficient of CFRP materials is 50% higher than that of GFRP materials [25]. Furthermore, the water absorption characteristics of C-GFRP materials may be influenced by the interface between CFRP and GFRP [26]. The water absorption behavior of C-GFRP rods have been investigated under conditions of water immersion [27], combined continuous bending, and water immersion [28]. The C-GFRP rod features a concentric circular cross-section comprising a CFRP inner layer and a GFRP outer layer, resulting in similar radial water absorption and diffusion behavior from the rod’s surface towards its core. Conversely, C-GFRP pipes consist of a thin inner CFRP layer and a thick outer GFRP layer, with both layers being exposed to hygrothermal environments. The water absorption behavior of CFRP exposure and GFRP exposure in a hygrothermal environment is different, and the degradation of mechanical properties is also different. Consequently, the current research findings are inadequate for application to C-GFRP winding pipes with a CFRP inner layer and a GFRP outer layer, particularly under varying hygrothermal environments inside and outside the pipes. Therefore, there is a pressing need to investigate the water absorption behavior of C-GFRP winding pipes, with a specific focus on the distinct effect of the inner and outer layers.

Transportation pipelines are subjected to various external loads, including pressure and impact. When these loads act in conjunction with the supports or brackets located at the pipeline base, specific requirements are imposed on the flexural properties [29,30]. The flexural properties of FRP materials have been revealed to be influenced by fiber type, resin, fiber volume content, the interfacial bonding strength between fibers and resin, etc. [31,32,33]. The ingress of moisture from hygrothermal environments can lead to property degradation, potentially resulting in flexural failure [34]. Previous studies have subjected carbon/epoxy (CE) and glass/epoxy (GE) composites to immersion in artificial seawater at 60 °C for 180 days. These investigations revealed that the degradation of tensile and shear strength is more pronounced in GE compared to CE, whereas the degradation of flexural properties is more severe in CE than in GF [35]. Additionally, there is evidence suggesting that the strength degradation of discontinuous CFRP immersed in seawater is more significant than that of continuous fibers [36]. Studies have indicated that, after immersing CFRP, GFRP, and C-GFRP plates in distilled water at 80 °C for 120 days, the retention rates of flexural strength are 87.00%, 74.17%, and 77.34%, respectively [37]. The low-velocity impact responses of C-GFRP pipes with GFRP layers on the outer and inner sides have been studied. Research indicates that the impact resistance of C-GFRP pipes with GFRP layers on the outer side is better [38], which proves the effect of fiber mixing species and locations on C-GFRP pipe properties. For C-GFRP pipes with a CFRP inner layer and a GFRP outer layer, when the bending direction is different, the materials in the tensile and compressive zones of the pipe are different, and the bending shapes of the cross-section are also different, resulting in different flexural properties [39,40]. It has been demonstrated that circular FRP pipes exhibit higher flexural strength and stiffness compared to square FRP pipes at an equivalent fiber winding angle. Consequently, circular FRP pipes are preferable for applications involving flexural loads [41]. Moreover, the differing durability characteristics of CFRP and GFRP materials in hygrothermal conditions add complexity to the evolution of the pipe’s flexural properties. Therefore, an experimental investigation is imperative to ascertain the evolution of flexural properties in C-GFRP pipes under different bending directions.

Through accelerated aging tests on FRP materials under hygrothermal conditions and the subsequent establishment of a correlation between environment and accelerated aging time, the long-term mechanical properties of FRP materials can be forecasted [42]. It has demonstrated that the time shift factor, based on Arrhenius theory, enables us to predicate life [43,44]. The adoption of the Arrhenius lifetime prediction model necessitates the assumption that the degradation mechanism of FRP remains consistent with increasing aging temperature and duration. Numerous accelerated aging tests have substantiated a linear relationship between the logarithm of aging time and the reciprocal of the temperature, thereby corroborating the assumptions of the Arrhenius theoretical model [41,45,46]. These findings reveal that, at lower temperatures, the degradation of FRP properties in distilled water environments is primarily attributed to resin hydrolysis and resin–fiber interface debonding. By employing higher accelerated aging temperatures, the experimental duration is shortened, allowing for the prediction of FRP lifespan at lower temperatures based on Arrhenius theory [25,42]. In previous research, CE and GE plates were subjected to immersion in an artificial seawater environment at 60 °C for 45 days, and the lifespan of their tensile strength was predicted utilizing Arrhenius theory. The findings indicate that the lifespan of CE significantly exceeds that of GE [47]. Subsequently, the plates were immersed for an extended duration of 180 days, and the tensile properties’ lifespan was again predicted based on Arrhenius theory. The results suggested that linear models provide sufficient accuracy for short-term life predictions, whereas nonlinear models are more appropriate for long-term life predictions [48,49]. Studies have indicated that the predicted lives of CFRP, GFRP, and C-GFRP plates with a three-point bending strength retention rate of 90% degradation at 23 °C are 361, 467, and 132 days, respectively [50]. However, the long-term performance of C-GFRP winding pipes with inner CFRP and outer GFRP layers, as well as GFRP in tension and GFRP in tension bending directions under complex working conditions, is not clear. Therefore, the applicability of Arrhenius theory to C-GFRP winding pipes remains to be rigorously validated.

In summary, the current research on the water absorption characteristics and flexural properties of FRP winding pipes is inadequate. Especially for special C-GFRP pipes with inner CFRP and outer GFRP layers, it is necessary to study the water absorption behavior of CFRP exposure and GFRP exposure in a distilled water environment, respectively, to accommodate the variable hygrothermal conditions inside and outside the pipes during service. Additionally, the flexural properties of GFRP in tension and GFRP in tension in hygrothermal conditions should be studied separately to account for the different materials and shapes of the pipe under different flexural directions. Moreover, it is necessary to predict the long-term lifetime of GFRP in tension and GFRP in tension in a hygrothermal environment and analyze the aging mechanism. The present study investigated the water absorption behavior and flexural properties’ evolution of C-GFRP winding pipes in an accelerated aging environment involving immersion in distilled water. The long-term flexural strength and life predictions for C-GFRP winding pipes were predicted based on Arrhenius theory. The results of this study are beneficial for promoting the long-term reliable application of C-GFRP winding pipes in oil, gas, and water pipeline transportation engineering.

## 2. Materials and Methods

### 2.1. Raw Materials

**Figure 1:**
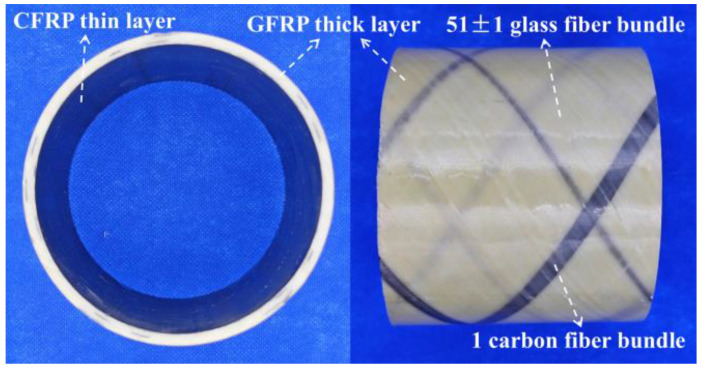
C/GFRP winding pipes.

The C-GFRP winding pipes were supplied by Shengli Xinda New Material Co., Ltd. (Dongying, China). The pipes were fabricated by initially forming an inner layer through the impregnation of carbon fiber fabric with an adhesive, with a thickness of 0.15 mm. Subsequently, 51 ± 1 glass fiber bundles along with one carbon fiber bundle, were impregnated and wrapped around the exterior of the inner layer. After the curing process, this assembly formed a C-GFRP winding pipe. The carbon fiber bundle was utilized for marking and inspection during the production process. The pipe exhibited a nominal wall thickness of 4 mm and a nominal outer diameter of 109 mm, as shown in Figure 1. The mass fractions were 2.74% for carbon fiber and 74.03% for glass fiber. The glass fiber employed was untwisted roving, while the resin utilized was bisphenol A epoxy resin, and the curing agent was an aromatic amine curing agent. The circumferential tensile strength of the C-GFRP winding pipe was measured to be 270.58 MPa, with a circumferential tensile modulus of 7.40 GPa and an elongation at break of 3.65%. The short-beam shear strength was 26.13 MPa. The glass transition temperature, determined by dynamic mechanical analysis (DMA) of the C-GFRP winding pipe, was 164.63 °C.

### 2.2. Exposure Conditions

An accelerated aging method was used to simulate the service hygrothermal conditions of C-GFRP pipes. The pipe samples were immersed in distilled water at 25 °C, 40 °C, and 60 °C to achieve accelerated aging in the laboratory. There are two primary rationales for utilizing a distilled water environment as a simulation environment. On the one hand, the external service environments of a C-GFRP winding pipe include marine environments, lake environments, groundwater environments, etc., and the internal transport solution include acids, alkali, salt, etc. The presence of water is a common characteristic in the various internal and external service environments of pipelines, so water environments cover a wider range of working conditions. On the other hand, previous studies have established that the aging of FRP in hygrothermal environments is mainly caused by resin hydrolysis and resin–fiber interface debonding [17,18].

### 2.3. Water Absorption Tests

**Figure 2:**
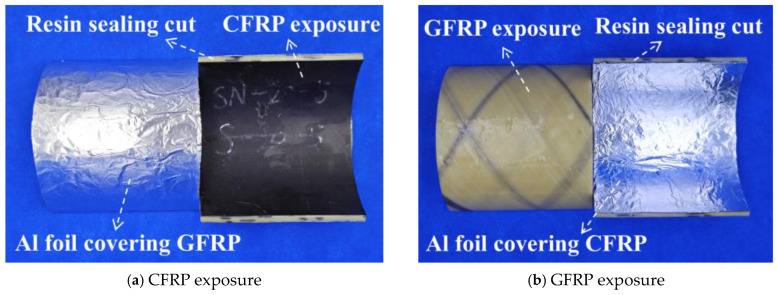
Water absorption sample.

To investigate the effects of hygrothermal conditions on the water absorption behavior of the inner and outer surfaces of C-GFRP winding pipes, water absorption tests were conducted on sliced samples. The dimension of each sample was 100 mm × 100 mm, according to ASTM D5229. The epoxy resin was applied to the cut around the samples to prevent direct moisture infiltration. Two single-sided water absorption pipe slice samples were prepared—one with the inner CFRP surface covered with aluminum foil to isolate it from water absorption (GFRP exposure) and the other with the outer-side GFRP surface covered to isolate it from water absorption (CFRP exposure)—thus allowing for controlled water absorption on either the inner CFRP surface or the outer GFRP surface, as shown in Figure 2. The samples were named using the following convention: water absorption surface (C and G represent CFRP exposure and GFRP exposure, respectively)—exposure condition temperature (T25, T40, and T60 represent 25 °C, 40 °C, and 60 °C, respectively), as detailed in Table 1.

An analytical balance was utilized to measure the mass of the pipe slice samples. The number of repeat samples was 5, and the results were averaged. The change percentage of water absorption for two types of samples was calculated, as shown in Equation (1).

(1) $$W_{t}=\frac{M_{t}-M_{0}}{M_{0}}$$

where t was the immersing time; $W_{t}$ was the water absorption at time t; $M_{0}$ was the initial weight of the sample; and $M_{t}$ was the weight of the sample at time t.

### 2.4. Three-Point Bending Tests

**Figure 3:**
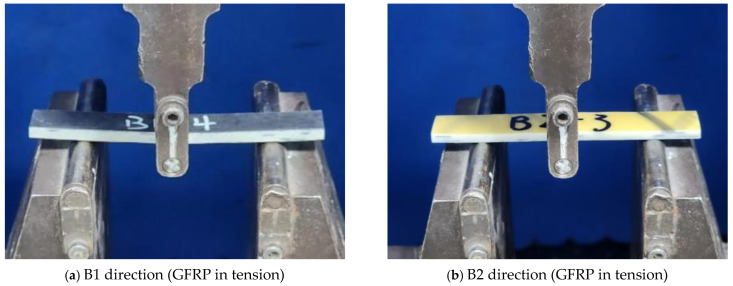
Three-point bending tests.

The three-point bending test was employed to assess the bending strength and modulus of C-GFRP winding pipes, as shown in Figure 3. According to ASTM D7264, the length of the bending sample was 100 mm, the outer width was 15 mm, the inner width was 14 mm, and the nominal thickness was 4.0 mm. The length direction of the sample was in the axial direction of the pipe. The three-point bending span was 64 mm, which corresponds to 16 times the sample thickness, with a loading rate of 10 mm/min. The loading device was the DHY-10080 universal testing machine, which was provided by Hengyi Precision Instrument Co., Ltd. (Shanghai, China). The loading was stopped until the sample underwent significant bending failure. Five repeat samples were tested for each condition, and the average values of the flexural strength and modulus were calculated.

To investigate the effect of different bending directions on the flexural properties of C-GFRP winding pipes, two bending directions were established. The first bending direction was the CFRP positioned vertically upward (GFRP in tension), and the second bending direction was the GFRP positioned vertically upward (CFRP in tension). Furthermore, to examine the effect of aging in distilled water at 25 °C, 40 °C, and 60 °C on the flexural properties of C-GFRP pipes, bending tests were performed on samples aged for 0 days, 30 days, 60 days, and 146 days in both bending directions. The bending samples were two-sided water absorption pipes. The samples were named according to the following convention: bending direction (B1 and B2 represent GFRP in tension and CFRP in tension, respectively)—immersing temperature (T25, T40, and T60 represent 25 °C, 40 °C, and 60 °C, respectively)—aging time (D0, D30, D60, and D146 represent 0 days, 30 days, 60 days, and 146 days, respectively), as shown in Table 2.

According to ASTM D7264 and the principles of material mechanics, the calculation equation for the flexural strength and modulus of a circular arc section with a thickness of 4.0 mm was derived, as shown in Equations (2) and (3), respectively.

(2) $$\sigma =\frac{2Fl\cdot y_{\max}}{(R_{1}^{4}-R_{2}^{4})\cdot (\theta +\sin\theta )-8y_{s}^{2}\cdot A}$$

(3) $$E=\frac{F\cdot l^{3}}{6\omega \cdot (R_{1}^{2}-R_{2}^{2})\cdot (\theta +\sin\theta )-48\omega \cdot y_{s}^{2}\cdot A}$$

where σ was the ultimate bending strength; F was the ultimate bearing capacity; l was the span; $y_{\max}$ was the farthest distance from the neutral axis to the edge; $R_{1}$ and $R_{2}$ were the outer diameter and inner diameter of the pipe; θ was the curvature of the sample; $y_{s}$ was the distance from the neutral axis to the center of the circle; A was the lateral area of the sample, which was equal to $(R_{1}^{2}-R_{2}^{2})\cdot \theta /2$; and ω was the linear segment deflection increment, mm.

## 3. Results and Discussion

### 3.1. Water Absorption Behavior

**Figure 4:**
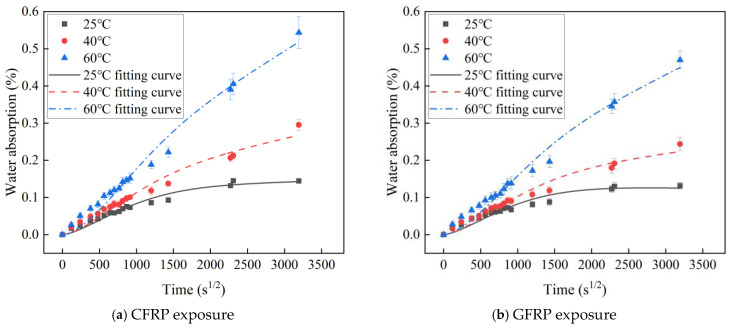
The water absorption and fitting curve of a C-GFRP winding pipe.

Figure 4 shows the water absorption behavior of C-GFRP winding pipe slices. The water absorption of C-GFRP winding pipes exhibits an increasing trend with temperature (at 25 °C, 40 °C, and 60 °C) and over time. After 146 days of immersion, the sample reaches its maximum water absorption. The water absorption of samples with CFRP exposure and GFRP exposure at the three temperatures demonstrate a two-stage change over time. The first stage is an initial rapid increase followed by a gradual decrease in the rate of absorption. Subsequently, the second stage is a linear increase. This behavior is consistent with a two-stage diffusion model [51], as shown in Equation (4). However, Equation (4) is only applicable to FRP sheets with two-sided water absorption. For sheets with single-sided water absorption, the thickness of the sample needs to be modified by Equation (5) to obtain the fitting function (Equation (6)). After 146 days of immersion, the water absorption process progresses into the second stage. Due to the effects of resin hydrolysis and debonding at the resin–fiber interface, moisture continues to infiltrate the sample until its structure is compromised, leading to a rapid increase in water absorption without reaching a discernible equilibrium point. Based on previous research on the two-stage water absorption model, the water absorption rate at the end of the first stage is considered the saturated water absorption rate, while no significant increase in the saturated water absorption rate is observed during the second stage [17]. The water absorption is fitted using Equation (6), and the results are presented in Figure 4 and Table 1.

As shown in Figure 4 and Table 1, the single-sided water absorption two-stage model demonstrates excellent concordance with the test results. The higher the temperature, the higher the maximum water absorption, saturated water absorption, and diffusion coefficient. This phenomenon can be attributed to the fact that, during the initial stage, water absorption is predominantly regulated by the concentration gradient of water molecules, adhering to Fick’s law of diffusion. Higher temperatures catalyze the moisture diffusion. Subsequently, in the second stage, the moisture induces relaxation of the resin polymer network, promotes resin hydrolysis, and disrupts resin–fiber interface bonding, thereby facilitating continued moisture infiltration [17].

(4) $$M_{t}=M_{\infty }\cdot \left(1+k\sqrt{t}\right)\left[1-\exp\left(-7.3{\left(\frac{Dt}{H^{2}}\right)}^{0.75}\right)\right]$$

(5) H=2h

(6) $$M_{t}=M_{\infty }\cdot \left(1+k\sqrt{t}\right)\left[1-\exp\left(-7.3{\left(\frac{Dt}{4h^{2}}\right)}^{0.75}\right)\right]$$

where k is a constant related to the relaxation of resin network structure and interfacial debonding; $M_{\infty }$ is the saturated water absorption; D is the diffusion coefficient; H is the thickness of the two-sided absorbent sample; and h is the thickness of the single-sided absorbent sample.

**Figure 5:**
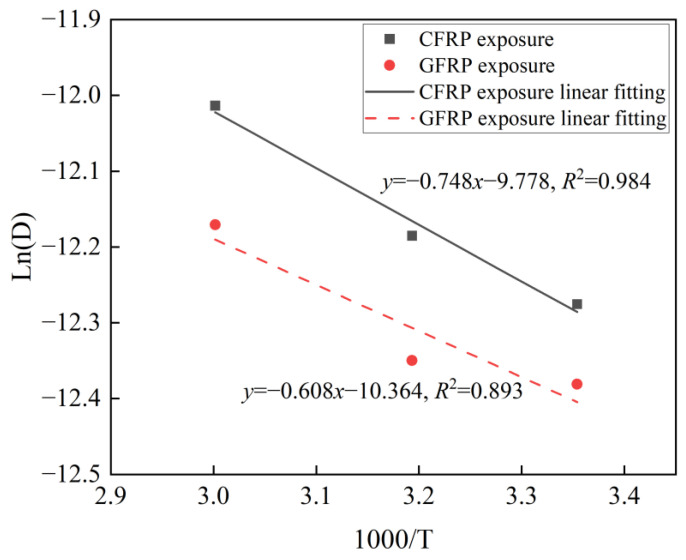
Effect of temperature on the water absorption diffusion coefficient.

The fitting relationship between water absorption and temperature was modeled using Arrhenius theory, as shown in Equation (7). Figure 5 shows the effect of temperature on the water absorption diffusion coefficient. A linear association between $Ln\left(D\right)$ and 1000/T is characterized by a slope with an absolute value of $E_{a}/R$ [52].

(7) $$\ln(D)=-\frac{E_{a}}{R}\cdot \frac{1}{T}+\ln(D_{0})$$

where D is the water absorption diffusion coefficient of the sample; $E_{a}$ is the activation energy; R is the universal gas constant; and T is the temperature in Kelvin.

A comparative analysis of the water absorption behavior between CFRP exposure and GFRP exposure provided the following conclusions: (1) In the first stage, saturated water absorption for CFRP exposure is 0.125% and that for GFRP exposure is 0.138%. The saturated water absorption adhering to Fick’s law in the first stage is 10.4% higher for GFRP exposure compared to for CFRP exposure. (2) The water absorption and diffusion coefficient for CFRP exposure are 11.19%, 18.01%, and 16.99% higher than those for GFRP exposure, at 25 °C, 40 °C, and 60 °C, respectively. This disparity can be attributed to the higher water absorption diffusion of the CFRP layer, coupled with the interface layer between the thin CFRP layer and the thick GFRP layer, which further enhances water absorption for CFRP exposure. (3) The activation energy is 6.22 kJ/mol for CFRP exposure and 5.05 kJ/mol on the GFRP side, with CFRP exposure exhibiting a 23.03% higher value compared to GFRP exposure. (4) After aging for 146 days, the water absorption for CFRP exposure is 9.09%, 20.90%, and 15.53% higher than that for GFRP exposure at 25 °C, 40 °C, and 60 °C, respectively. This difference is attributed to the higher diffusion coefficient and activation energy observed for CFRP exposure.

### 3.2. Evolution Law of Flexural Properties

#### 3.2.1. Flexural Failure Mode

**Figure 6:**
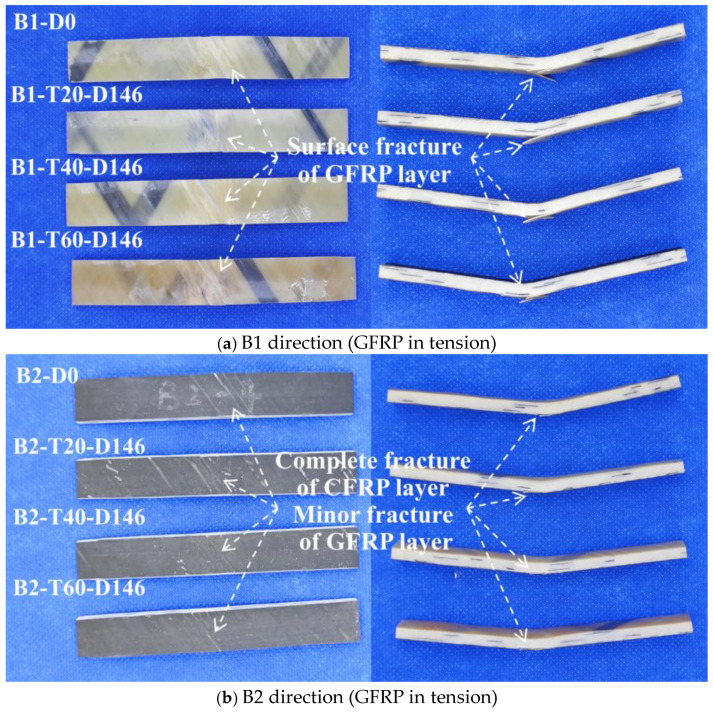
Flexural failure mode.

Figure 6 shows the flexural failure modes observed in the specimens tested in the B1 and B2 directions. It reveals evident flexural failure in both the B1 and B2 directions. Specifically, the B1 direction exhibits fracture failure on the surface of the GFRP layer, while the B2 direction exhibits fracture failure for both the whole CFRP layer and the minor GFRP layer. Figure 6 shows that after immersion and aging in distilled water for 146 days, the surface color of the 60 °C sample turned dark yellow and the surface color of the 40 °C sample turned light yellow. This indicates that there is a significant aging layer on the surface of both the 40 °C and 60 °C samples, with the 60 °C sample displaying a more pronounced aging effect compared to the 40 °C sample. The thickness of the 60 °C sample increases due to the higher water absorption rate caused by the higher temperature, resulting in volume expansion of the sample after absorbing moisture. Notably, the failure modes of the sample at 25 °C, 40 °C, and 60 °C remained unchanged compared to their initial states.

#### 3.2.2. Flexural Strength

**Figure 7:**
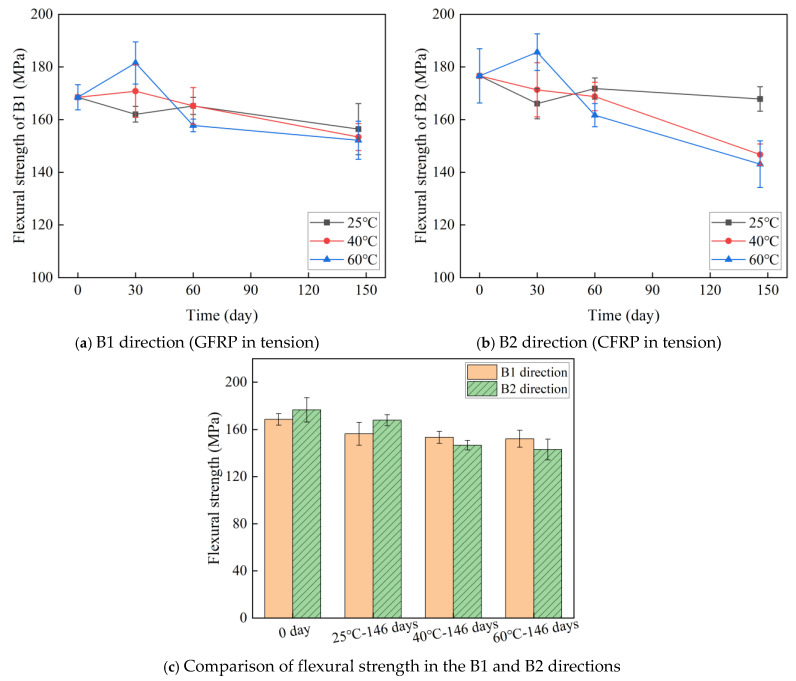
The effect of temperature and time on the flexural strength.

Figure 7 and Table 2 show the effect of temperature and time on the flexural strength of C-GFRP winding pipes. The following conclusions are drawn:

(1) The initial flexural strength of the C-GFRP winding pipe is 168.49 MPa in the B1 direction and 176.60 MPa in the B2 direction, with the B2 direction exhibiting a 4.81% higher flexural strength. This discrepancy can be attributed to the CFRP layer in the B2 direction reinforcing the tensile zone of the pipe during the bending process.
(2) After aging for 30 days, the flexural strength of the B1 direction at 25 °C decreased by 3.83%, increased by 1.37% at 40 °C, and increased by 7.73% at 60 °C. The flexural strength at 25 °C in the B2 direction decreased by 5.96%, decreased by 3.00% at 40 °C, and increased by 5.09% at 60 °C. The observed improvement in properties can be explained by the incomplete curing of the resin inside the pipe, which was further cured under a heating aging environment [53,54]. The highest temperature of 60 °C resulted in the most pronounced post-curing effect and subsequent property enhancement.
(3) After 60 days of aging, the flexural strength in the B1 direction increased by 1.96% at 25 °C, decreased by 3.26% at 40 °C, and decreased by 13.07% at 60 °C compared to after 30 days. The flexural strength at 25 °C in the B2 direction increased by 3.44%, decreased by 1.48% at 40 °C, and decreased by 12.87% at 60 °C. This trend can be attributed to the delayed strength improvement caused by post-curing at the lower temperature of 25 °C. While at higher temperatures (40 °C and 60 °C), the post-curing effect diminishes, and the properties are primarily governed by resin hydrolysis and interfacial debonding, leading to a significant reduction in flexural strength.
(4) After 146 days of aging, the flexural strength of the B1 and B2 directions showed significant degradation compared to the initial strength, as shown in Figure 7c. The flexural strength of the B1 direction at 25 °C, 40 °C, and 60 °C decreased by 7.18%, 8.96%, and 9.70%, respectively. The flexural strength at 25 °C, 40 °C, and 60 °C in the B2 direction decreased by 4.97%, 16.9%, and 18.97%, respectively. This indicates that as the water absorption of the samples increases, the resin hydrolysis and interfacial debonding in the pipes intensify, leading to further deterioration of the flexural strength.
(5) After aging for 146 days, the flexural strength in the B2 direction at 40 °C and 60 °C was 5.95% lower than that in the B1 direction. This discrepancy can be attributed to the higher diffusion coefficient of the CFRP layer relative to the internal GFRP layer. Additionally, the interfacial layer between the CFRP and GFRP layers further enhances water absorption within the CFRP side. Consequently, the CFRP side exhibits greater water absorption than the GFRP side, leading to more pronounced resin hydrolysis and interface debonding, ultimately resulting in a more significant reduction in bending strength [55]. These findings are in accordance with the water absorption results obtained for CFRP exposure and GFRP exposure, as shown in Table 1.

#### 3.2.3. Flexural Modulus

**Figure 8:**
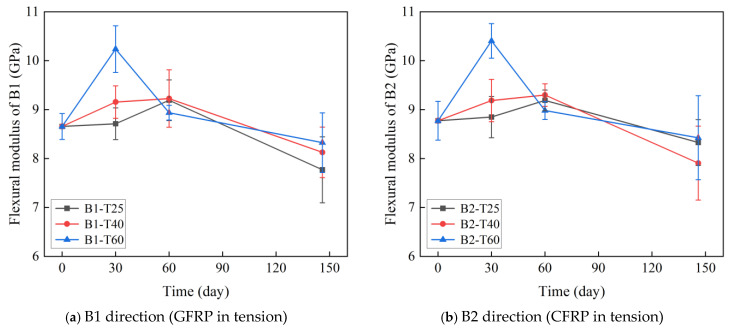
The effect of temperature and time on the flexural modulus.

The effect of temperature and time on the flexural modulus of C-GFRP winding pipes are shown in Figure 8 and Table 3. As shown in the figure, the initial flexural modulus in the B1 direction (8.66 GPa) and B2 direction (8.77 GPa) are similar in magnitude.

The evolution of the flexural modulus in both the B1 and B2 directions of the C-GFRP winding pipes follows a similar trend. (1) After aging for 30 days, the flexural modulus in both the B1 and B2 directions increased, which can be explained by the incomplete curing of the resin, resulting in post-curing after exposure to the heating environment. (2) After aging for 60 days, the flexural modulus in the B1 and B2 directions continued to increase at 25 °C and 40 °C, indicating that the post-curing effect is not complete in the two lower temperatures. Conversely, at 60 °C, the flexural modulus in both directions decreased, indicating a diminution of the post-curing effect and a notable reduction in flexural stiffness due to resin hydrolysis and interfacial debonding. (3) After aging for 146 days, the flexural modulus in the B1 and B2 directions decreased at 25 °C, 40 °C, and 60 °C. This indicates that the bending stiffness was predominantly influenced by resin hydrolysis and interfacial debonding.

### 3.3. Long-Term Life Prediction of Flexural Strength

#### 3.3.1. Life Prediction Model

Based on the Arrhenius prediction model, accelerated testing is conducted in distilled water conditions at temperatures of 25 °C, 40 °C, and 60 °C to predicate the long-term flexural strength retention rate of C-GFRP winding pipes. The detailed prediction procedure is as follows:

Firstly, Equation (8) is employed to fit the strength retention rate of C-GFRP winding pipes immersed in distilled water conditions at temperatures of 25 °C, 40 °C, and 60 °C [56], and the fitting parameters τ are obtained.

(8) Y=100exp(−t/τ)

where Y is the flexural strength retention rate; t is the aging time; and τ is the fitting parameter.

Subsequently, based on the Arrhenius prediction model, the relationship between the degradation rate and aging temperature of C-GFRP winding pipes can be expressed as Equation (9) [50].

(9) $$k=A\exp(-E_{a}/RT)$$

where k is the degradation rate of flexural strength; A is the degradation constant; and $E_{a}$ is the activation energy.

By taking the reciprocal of both sides of Eqution (9), Equation (10) is derived:

(10) $$\frac{1}{k}=\frac{1}{A}\exp(E_{a}/RT)$$

Taking the e logarithm of both sides of Equation (10) yields Equation (11):

(11) $$\ln(\frac{1}{k})=\frac{E_{a}}{R}\frac{1}{T}-\ln A$$

According to Equation (11), a linear correlation exists between ln(1/k) and 1/T, with a slope of $E_{a}/R$.

Furthermore, the aging time for the strength retention rate to decrease to the same value at an accelerated test temperature $T_{0}$ and a given predicted environmental temperature $T_{1}$ are defined as $t_{0}$ and $t_{1}$, respectively. Based on Equation (9), the ratio of $t_{1}$ to $t_{0}$ is established as the time shift factor (TSF), leading to Equation (12):

(12) $$TSF=\frac{t_{0}}{t_{1}}==\frac{k_{1}}{k_{0}}=\frac{A\exp(-E_{a}/RT_{1})}{A\exp(-E_{a}/RT_{0})}=\exp\left[\frac{E_{a}}{R}\left(\frac{1}{T_{0}}-\frac{1}{T_{1}}\right)\right]$$

The fitting parameters obtained from Equation (8) are then substituted back into Equation (8) to determine the time required for the flexural strength retention rate of C-GFRP winding pipes to decline to 80%, 85%, 90%, and 95% at 25 °C, 40 °C, and 60 °C. Equation (11) is used to fit the relationships between four sets of ln(1/k) and group 1/T and to obtain four sets of slopes $E_{a}/R$.

Subsequently, the TSF between $T_{1}$ and $T_{0}$ is predicted using Equation (12). Finally, the flexural strength retention rates at 25 °C, 40 °C, and 60 °C are multiplied TSF and fitted using Equation (8) to establish the mechanical property evolution curve of the flexural strength retention rate at the given predicted environmental temperature. This curve serves as a life prediction curve for the C-GFRP winding pipes.

#### 3.3.2. Flexural Strength Retention Rate Fitting Curve

**Figure 9:**
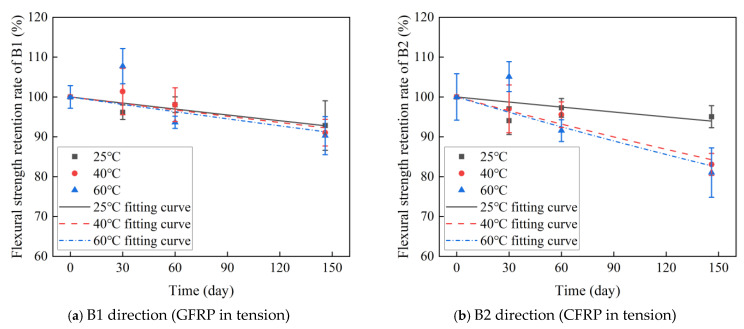
Fitting results of the flexural strength retention of a C-GFRP winding pipe.

**Figure 10:**
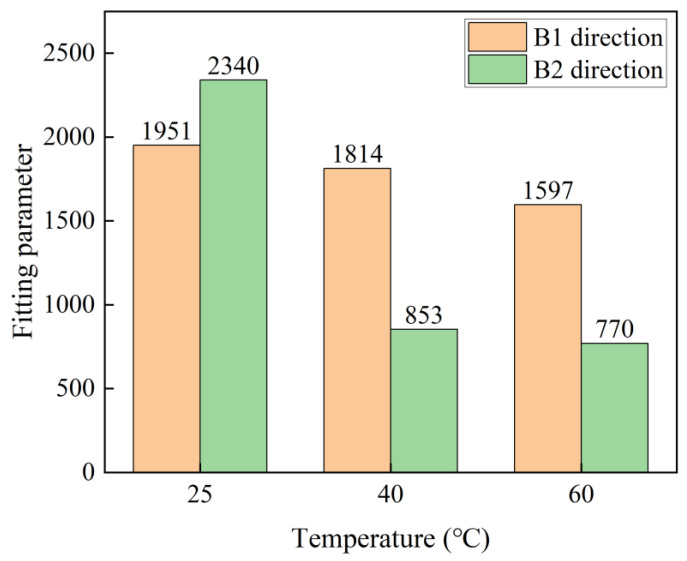
The fitting parameters of flexural strength retention.

Based on Equation (8), the evolution of the flexural strength retention rate in the B1 and B2 directions of C-GFRP winding pipes is modeled, with the fitting parameters τ presented in Table 4. Figure 9 shows the fitting results of the flexural strength retention of a C-GFRP winding pipe. Figure 10 shows the fitting parameters of the flexural strength retention of a C-GFRP winding pipe. Specifically, the fitting parameters for temperatures of 25 °C, 40 °C, and 60 °C in the B1 direction are 1951, 1814, and 1957, respectively. The fitting parameters for temperatures of 25 °C, 40 °C, and 60 °C in the B1 direction are 2340, 853, and 770, respectively. Notably, the fitting parameters exhibit a decreasing trend with rising temperature, being highest at 60 °C, followed by 40 °C, and lowest at 25 °C. This suggests that an increase in aging temperature leads to a reduction in both the fitting parameters and the flexural strength of the C-GFRP winding pipes. Furthermore, the flexural strength retention rate in the B1 direction is consistently higher than that in the B2 direction at 20 °C and 40 °C, resulting in lower fitting parameters for the B2 direction compared to the B1 direction.

#### 3.3.3. Arrhenius Theoretical Life Prediction

**Figure 11:**
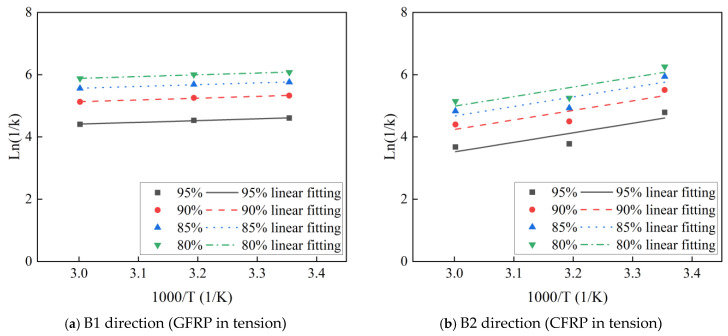
The Arrhenius linear fitting of the flexural strength.

**Figure 12:**
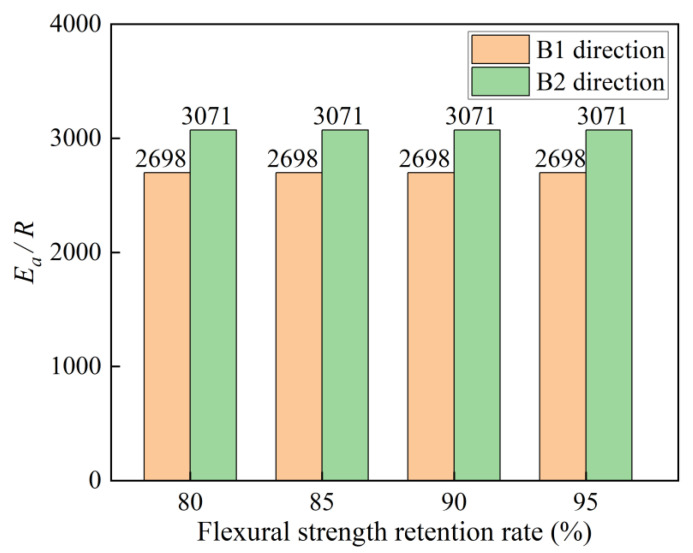
The relationship between the fitting parameters and flexural strength retention rate.

By substituting τ from Table 4 into Equation (10), the time is calculated for the flexural strength retention rates of C-GFRP winding pipes to decrease to 80%, 85%, 90%, and 95% at temperatures of 25 °C, 40 °C, and 60 °C. Subsequently, the linear fittings are conducted utilizing Equation (11), with the parameters of linear fitting presented in Figure 11 and Table 5. The fitting accuracy of the curves for both the B1 and B2 directions are notably high, and the $E_{a}/R$ remain consistent across all four strength retention rates. Figure 12 shows the relationship between the fitting parameters $E_{a}/R$ and flexural strength retention rate. The $E_{a}/R$ derived from the fitting line for the B1 and B2 directions are 2698 and 3071, respectively. This suggests that the flexural strength degradation curves are well suited for long-term life prediction models based on Arrhenius theory.

**Figure 13:**
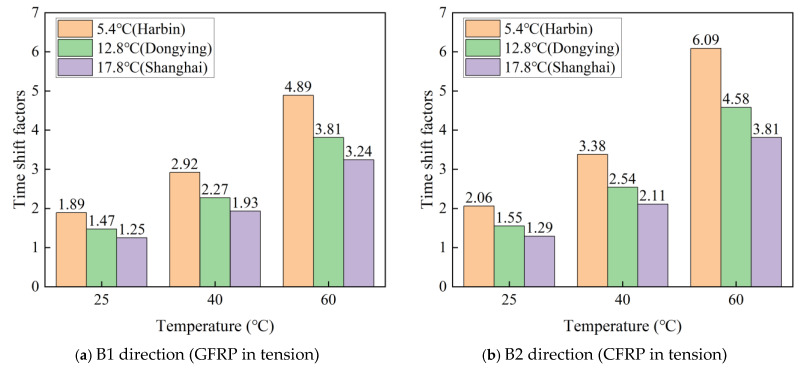
The time shift factors between the given predicted environmental temperatures and accelerated test temperatures.

Utilizing monitoring data obtained from the China Meteorological Administration, the average temperatures of the Chinese cities Harbin, Dongying, and Shanghai for the year 2023 are 5.4 °C, 12.8 °C, and 17.8 °C, respectively. The time shift factors (TSF) between the three given predicted environmental temperatures and three accelerated test temperatures of 25 °C, 40 °C, and 60 °C are calculated in Equation (12). The calculated time shift factors in the B1 and B2 directions are listed in Table 6. Figure 13 shows the time shift factors (TSF) between the given predicted environmental temperatures and accelerated test temperatures. In this life prediction model, the time shift factors decrease with increasing given predicted environmental temperatures and increase with increasing accelerated test temperatures. Under the same given predicted environmental temperatures and increasing accelerated test temperatures, the time shift factors are higher in the B2 bending direction than in the B1 direction.

**Figure 14:**
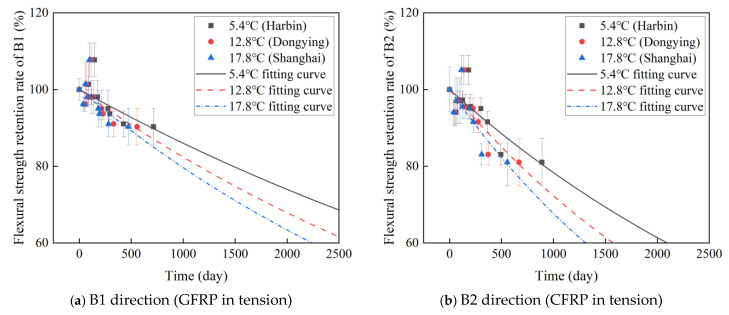
Long-term life prediction curves of C-GFRP winding pipes at given temperatures.

**Figure 15:**
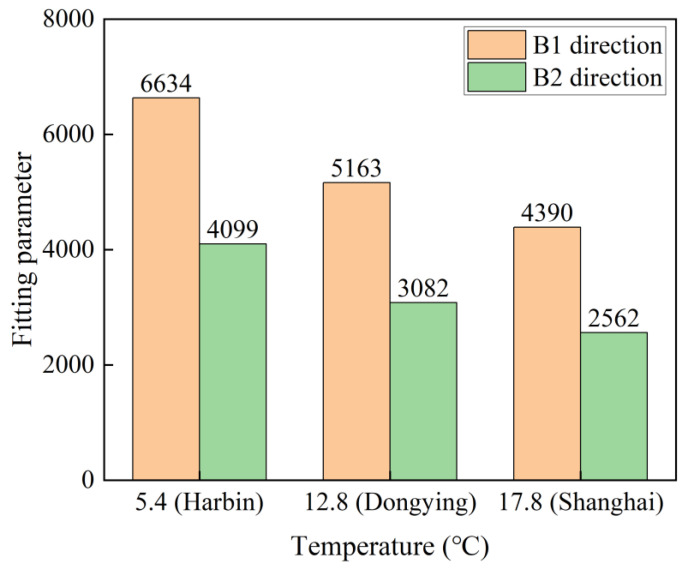
The fitting parameters between the given predicted environmental temperatures and accelerated test temperatures.

By overlaying the curve depicted in Figure 9 with the corresponding data from Table 6, the flexural strength retention rates of C-GFRP pipes at 5.4 °C, 12.8 °C, and 17.8 °C are derived. Subsequently, the long-term life prediction curve is fitted using Equation (8), as shown in Figure 14, with the fitting parameters listed in Table 7. Figure 15 shows the effect of the given predicted environmental temperatures on the fitting parameters. The fitting parameters decrease with increasing given predicted environmental temperatures. In the same given predicted environmental temperatures, the fitting parameters in the B2 direction are lower than those in the B1 direction.

As shown in Figure 9, the degradation rate of flexural strength for C-GFRP winding pipes is slowest at 5.4 °C (Harbin), followed by 12.8 °C (Dongying), and most rapid at 17.8 °C (Shanghai) in both the B1 and B2 directions. A comparison of the degradation rates in the B1 and B2 directions reveals that the rate in the B2 direction exceeds that in the B1 direction. These findings are in good agreement with the test results obtained under accelerated aging conditions in the laboratory.

**Figure 16:**
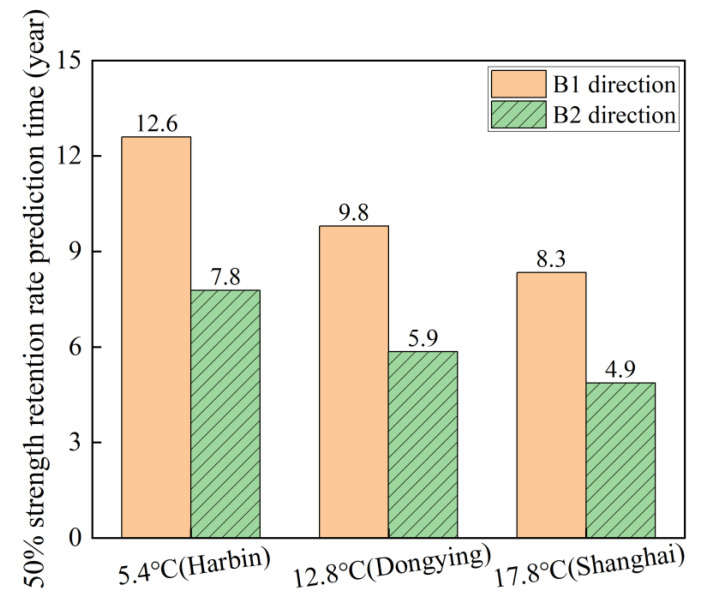
A comparison of life prediction in the B1 and B2 directions.

Finally, the aging time corresponding to a 50% retention rate [45] of flexural strength under the given predicted environmental exposure was adopted as the service life metric. Based on the fitting results presented in Figure 14, the service life of flexural strength degradation for B1 and B2 to 50% in Harbin (5.4 °C), Dongying (12.8 °C), and Shanghai (17.8 °C) is determined, as shown in Figure 16 and listed in Table 8. Specifically, in the B1 direction, the corresponding aging times for Harbin, Dongying, and Shanghai are 12.60 years, 9.80 years, and 8.34 years, respectively. In the B2 direction, the corresponding aging times are 7.78 years, 5.85 years, and 4.87 years, respectively. The predicted service lives in the B1 direction are 61.95% (Harbin), 67.52% (Dongying), and 71.25% (Shanghai) longer than that in the B2 direction. It is noteworthy that, according to existing research findings, the predicted lifespan under the accelerated aging conditions of distilled water immersion in the laboratory is highly conservative. This is attributed to two primary factors: firstly, the sealed environment of distilled water in the laboratory exacerbates the degradation of resin hydrolysis products [57]. Secondly, in service conditions, pipelines are not subject to long-term water immersion both internally and externally. Consequently, the actual lives are expected to exceed the predicted lives.

## 4. Conclusions

In this study, carbon–glass hybrid fiber-reinforced epoxy polymer (C-GFRP) winding pipes were subjected to accelerated aging by immersion in distilled water at 25 °C, 40 °C, and 60 °C for 146 days. The water absorption behavior of the C-GFRP winding pipes was investigated through water absorption tests, while their flexural strength and modulus evolution were studied through bending tests. The long-term flexural strength and life of the C-GFRP winding pipes were predicted based on Arrhenius theory. The findings of this study are summarized as follows:

(1) The single-sided water absorption behavior of C-GFRP winding pipes follows a two-stage water absorption model. The water absorption increases with temperature and time. In the first stage, the water absorption curve follows Fick’s law. In the second stage, the moisture induces resin hydrolysis and interface bonding. After aging for 146 days, the water absorption, diffusion coefficient, and activation energy of the CFRP exposure are higher than those for GFRP exposure.
(2) The flexural strength and modulus of C-GFRP winding pipes are affected by the combined effects of post-curing, resin hydrolysis, and debonding. During the early stages of aging, C-GFRP pipes at 40 °C and 60 °C experienced an increase in flexural strength modulus due to post-curing effects. After aging for 140 days, resin hydrolysis and resin–fiber interface debonding become dominant, leading to a degradation in flexural strength and modulus, with the flexural strength retention rate decreasing as temperature increases.
(3) Initially, the flexural strength of C-GFRP winding pipes in the B1 direction (GFRP in tension) is lower than that in the B2 direction (CFRP in tension). However, after aging for 140 days, the flexural strength in the B1 direction becomes higher than that in the B2 direction. Over time, the higher water absorption and diffusion coefficient of the CFRP layer, coupled with the interface layer between the CFRP and GFRP, exacerbated resin hydrolysis and interfacial debonding in the B1 direction.
(4) Based on Arrhenius theory, the long-term life prediction of the flexural strength retention rate of C-GFRP winding pipes degraded to 50% was carried out. The B1 direction long-term lives of flexural strength in Harbin (5.4 °C), Dongying (12.8 °C), and Shanghai (17.8 °C) are 12.60 years, 9.80 years, and 8.34 years, respectively, while the B2 direction long-term lives are 7.78 years, 5.85 years, and 4.87 years, respectively.

## Figures and Tables

**Table 1:** The water absorption fitting parameters of a C-GFRP winding pipe.

Exposure Side	Sample	Temperature	$M_{\max}$/%	$M_{\infty }$/%	D/10^−6^ m^2^/s	$R^{2}$
CFRP exposure	C-T25	25 °C	0.144	0.125	4.67	0.963
	C-T40	40 °C	0.295	0.125	5.11	0.965
	C-T60	60 °C	0.543	0.125	6.06	0.980
GFRP exposure	G-T25	25 °C	0.132	0.138	4.20	0.933
	G-T40	40 °C	0.244	0.138	4.33	0.962
	G-T60	60 °C	0.470	0.138	5.18	0.978

**Table 2:** The effect of temperature and time on the flexural strength.

Loading Direction	Temperature/°C	Sample	Aging Time /Day	Flexural Strength /MPa	Standard Deviation/MPa	Retention Rate /%	Standard Deviation /%
B1 direction (GFRP in tension)	25	B1-T25-D0	0	168.49	4.78	100.00	2.84
		B1-T25-D30	30	162.03	2.96	96.17	1.83
		B1-T25-D60	60	165.20	3.27	98.05	1.98
		B1-T25-D146	146	156.40	9.71	92.83	6.21
	40	B1-T40-D0	0	168.49	4.78	100.00	2.84
		B1-T40-D30	30	170.79	9.93	101.37	5.82
		B1-T40-D60	60	165.22	7.00	98.06	4.24
		B1-T40-D146	146	153.39	5.14	91.04	3.35
	60	B1-T60-D0	0	168.49	4.78	100.00	2.84
		B1-T60-D30	30	181.52	8.00	107.74	4.41
		B1-T60-D60	60	157.79	2.40	93.65	1.52
		B1-T60-D146	146	152.15	7.25	90.30	4.76
B2 direction (CFRP in tension)	25	B2-T25-D0	0	176.60	10.30	100.00	5.83
		B2-T25-D30	30	166.08	5.76	94.05	3.47
		B2-T25-D60	60	171.80	4.02	97.28	2.34
		B2-T25-D146	146	167.83	4.64	95.04	2.77
	40	B2-T40-D0	0	176.60	10.30	100.00	5.83
		B2-T40-D30	30	171.31	10.26	97.01	5.99
		B2-T40-D60	60	168.77	5.41	95.57	3.21
		B2-T40-D146	146	146.71	4.05	83.08	2.76
	60	B2-T60D-0	0	176.60	10.30	100.00	5.83
		B2-T60-D30	30	185.59	6.96	105.09	3.75
		B2-T60-D60	60	161.70	4.41	91.56	2.73
		B2-T60-D146	146	143.10	8.85	81.03	6.19

**Table 3:** The effect of temperature and time on the flexural modulus.

Loading Direction	Temperature/°C	Sample	Aging Time /Day	Flexural Modulus /MPa	Standard Deviation/MPa
B1 direction (GFRP in tension)	25	B1-T25-D0	0	8.66	0.26
		B1-T25-D30	30	8.71	0.32
		B1-T25-D60	60	9.19	0.42
		B1-T25-D146	146	7.77	0.68
	40	B1-T40-D0	0	8.66	0.26
		B1-T40-D30	30	9.16	0.33
		B1-T40-D60	60	9.23	0.59
		B1-T40-D146	146	8.13	0.52
	60	B1-T60-D0	0	8.66	0.26
		B1-T60-D30	30	10.24	0.48
		B1-T60-D60	60	8.94	0.15
		B1-T60-D146	146	8.32	0.61
B2 direction (CFRP in tension)	25	B2-T25-D0	0	8.77	0.40
		B2-T25-D30	30	8.85	0.42
		B2-T25-D60	60	9.19	0.21
		B2-T25-D146	146	8.33	0.47
	40	B2-T40-D0	0	8.77	0.40
		B2-T40-D30	30	9.19	0.43
		B2-T40-D60	60	9.30	0.23
		B2-T40-D146	146	7.91	0.76
	60	B2-T60D-0	0	8.77	0.40
		B2-T60-D30	30	10.41	0.36
		B2-T60-D60	60	8.98	0.18
		B2-T60-D146	146	8.43	0.86

**Table 4:** Fitting results of the flexural strength retention of a C-GFRP winding pipe.

Loading Direction	Temperature/°C	Fitting Parameter
B1 direction (GFRP in tension)	25	1951
	40	1814
	60	1597
B2 direction (CFRP in tension)	25	2340
	40	853
	60	770

**Table 5:** Fitting parameters obtained by Arrhenius linear fitting.

Loading Direction	Flexural Strength Retention Rate/%	$E_{a}/R$	$R^{2}$
B1 direction (GFRP in tension)	80	2698	0.99
	85	2698	0.99
	90	2698	0.99
	95	2698	0.99
B2 direction (CFRP in tension)	80	3071	0.78
	85	3071	0.78
	90	3071	0.78
	95	3071	0.78

**Table 6:** Time shift factors of C-GFRP winding pipes at different temperatures.

Loading Direction	Temperature/°C	5.4 °C (Harbin)	12.8 °C (Dongying)	17.8 °C (Shanghai)
B1 direction (GFRP in tension)	25	1.89	1.47	1.25
	40	2.92	2.27	1.93
	60	4.89	3.81	3.24
B2 direction (CFRP in tension)	25	2.06	1.55	1.29
	40	3.38	2.54	2.11
	60	6.09	4.58	3.81

**Table 7:** The fitting parameters of the long-term life prediction curves.

Loading Direction	Predicted Region	Temperature/°C	Fitting Parameter τ
B1 direction (GFRP in tension)	Harbin	5.4	6634
	Dongying	12.8	5163
	Shanghai	17.8	4390
B2 direction (CFRP in tension)	Harbin	5.4	4099
	Dongying	12.8	3082
	Shanghai	17.8	2562

**Table 8:** The service lives of flexural strength degradation to 50%.

Loading Direction	Predicted Region	Average Temperature in 2023	Prediction Life /Year
B1 direction (GFRP in tension)	Harbin	5.4 °C	12.60
	Dongying	12.8 °C	9.80
	Shanghai	17.8 °C	8.34
B2 direction (CFRP in tension)	Harbin	5.4 °C	7.78
	Dongying	12.8 °C	5.85
	Shanghai	17.8 °C	4.87

## Data Availability

The original contributions presented in this study are included in the article. Further inquiries can be directed to the corresponding author.
